# Development and validation of diagnostic SNP markers for quality control genotyping in a collection of four rice (Oryza) species

**DOI:** 10.1038/s41598-021-97689-3

**Published:** 2021-09-20

**Authors:** Arnaud Comlan Gouda, Marilyn L. Warburton, Gustave L. Djedatin, Sèdjro Bienvenu Kpeki, Peterson W. Wambugu, Karlin Gnikoua, Marie Noelle Ndjiondjop

**Affiliations:** 1 Africa Rice Center (AfricaRice), M’bé Research Station, 01 B.P. 2551, Bouaké 01, Côte d’Ivoire; 2 United States Department of Agriculture-Agricultural Research Service, Corn Host Plant Resistance Research Unit, Mississippi State, USA; 3 Université Nationale des Sciences, Technologies, Ingénierie et Mathématiques (UNSTIM), Abomey, Benin; 4 Kenya Agricultural and Livestock Research Organization (KALRO), Genetic Resources Research Institute, Nairobi, Kenya

**Keywords:** Molecular biology, Plant sciences

## Abstract

Morphological identification of closely related rice species, particularly those in the *Oryza* AA genome group, presents major challenges and often results in cases of misidentification. Recent work by this group identified diagnostic single nucleotide polymorphic (SNP) markers specific for several rice species and subspecies based on DArTseq next-generation sequencing technology (“DArTseq”). These SNPs can be used for quality control (QC) analysis in rice breeding and germplasm maintenance programs. Here, we present the DArTseq-based diagnostic SNPs converted into Kompetitive allele-specific PCR (KASPar or KASP) assays and validation data for a subset of them; these can be used for low-cost routine genotyping quality control (QC) analysis. Of the 224 species/subspecies’ diagnostic SNPs tested, 158 of them produced working KASP assays, a conversion success rate of 70%. Two validation experiments were run with 87 of the 158 SNP markers to ensure that the assays amplified, were polymorphic, and distinguished the five species/subspecies tested. Based on these validation test results, we recommend a panel of 36 SNP markers that clearly delineate *O. barthii, O. glaberrima*, *O. longistaminata*, *O. sativa* spp. *indica* and *japonica*. The KASP assays provide a flexible, rapid turnaround and cost-effective tool to facilitate germplasm curation and management of these four *Oryza* AA genome species across multiple genebanks.

## Introduction

Rice belongs to the genus *Oryza*, which consists of 27 species and 11 genome types^1^, of which, eight species, representing five genomes, are found in Africa. This includes the AA (*Oryza barthii* A. Chev., *O. glaberrima* Steud., *O. sativa* L., and *Oryza longistaminata* Chev. and Röhr); BB (*O. punctata*); CC (*O. eichingeri*); FF (*O. brachyantha*); and BBCC (*O. schweinfurthiana*) genome species^2^. The four AA genome *Oryza* species account for nearly 99% of the ~ 21,300 rice accessions conserved at the AfricaRice genebank (https://www.genesys-pgr.org/). *O. glaberrima* is taxonomically differentiated from *O. barthii* using phenotypic characters, such as growth habit, spikelet shattering, and hairiness of awn and spikelets. However, it is often difficult to clearly separate these two indigenous African species based on phenotype for a few different reasons: (i) *O. glaberrima* is thought to have evolved from *O. barthii* through selection^3,4^ and both species are highly similar even at the molecular level^5^; (ii) the phenotypic traits used to differentiate the two species are not conclusive across diverse accessions and are highly affected by environment and/or ecology; and (iii) there is a wide range of intermediate types between *O. barthii* and *O. glaberrima* that are much more challenging to differentiate from the two species^4^.

The two *O. sativa* subspecies, *indica* and *japonica*, are also taxonomically classified based on their morphological traits (number of tillers, plant height, plant type, plant pubescence, and type/length of grains) in combination with other traits (including winter hardiness, starch types and phenol response in grains)^6^. However, the phenotypic attributes used to differentiate *indica* from *japonica* have low resolution to clearly separate many collections, are laborious to measure and are highly affected by environmental conditions. Since the advent of molecular marker technology, the phylogenetic relationships among the *Oryza* species have been determined using both low-density and high-density molecular markers^1,6–8^. Recently, our team at the AfricaRice reported 339 diagnostic single nucleotide polymorphic (SNP) markers that can be used for accurate taxonomic classification of the *O. barthii, O. glaberrima*, *O. longistaminata* and the two *O. sativa* subspecies^5,9^. Validation of potential use of these markers for breeding and germplasm maintenance in these species and subspecies forms the basis of the current study.

The conservation of plant genetic resources began as a response to the rapid loss of agricultural biodiversity mainly due to continuous replacement of traditional varieties (landraces) by modern varieties, changes in eating habits, and rampant development, which has threatened local landraces and crop wild relatives^10^. Genebanks were established as repositories to safeguard plant genetic resources for food and agriculture (PGRFA) against loss, and serve as a critical source of genes for improving agricultural crops^11^. Currently, there are nearly 7.4 million plant germplasm entries (accessions) conserved by numerous institutions^12^ of which, the CGIAR System Organization (CGIAR) genebanks hold 21% of the global ex situ rice collections (https://www.genesys-pgr.org/). AfricaRice is one of the 11 CGIAR institutions that have genebanks intended for the conservation of a wide range of germplasm types (wild, weedy, traditional varieties/landraces, breeding/research materials, and advanced/improved cultivars). The CGIAR genebanks make these PGRFA available under the terms of the International Treaty on Plant Genetic Resources for Food and Agriculture and distribute them to the global community free of charge.

Some of the major challenges facing genebank managers and plant genetic resource scientists^13^ include taxonomic misclassification (misidentification or misnaming) and mislabeling, which lead to errors in various genebank operations^5,14,15^. These errors negatively affect effective conservation, dissemination and use of germplasm^16,17^. Most taxonomic misclassification and mislabeling are due to human error during planting of material, characterization of accessions for phenotypic traits, and mislabeling or misreading of the germplasm names^18^ during the various genebank operations. Such errors have been reported in various crop species and error rates vary from 3 to 28%^5,18–22^. Recently, the impact of taxonomic misnaming was highlighted in a study of the genus *Citrullus* (watermelon) in two major databases: Genesys PGR (https://www.genesys-pgr.org/) and the European Search Catalogue for Plant Genetic Resources (EURISCO, https://eurisco.ipk-gatersleben.de/^15^). Just 3% of the *Citrullus* accessions for which information is stored in these two databases was found to have been correctly named, and 28% of the collections showed major taxonomic errors. As a consequence, over a quarter of the *Citrullus* collection has never been used in research, breeding, cultivation, and reintroduction projects. These types of errors can best be avoided (or at least minimized) by implementing routine genotyping quality control (QC) methods using low cost, high throughput and user-friendly SNP markers.

The numerous SNP genotyping platforms may be divided into (a) genotyping-by-sequencing (GBS) methods based on next generation sequencing technologies; (b) those platforms that use fixed SNP assays; and (c) SNP assays that run one marker at a time (uniplex), usually PCR based^23,24^. Next generation sequencing technologies-based platforms, such as GBS^25^ and Diversity Array technology-based sequencing (DArTseq)^26^ have been found to be cost-effective and rapid for applications that require high-density markers, such as germplasm characterization, gene discovery and genomic selection. Other applications such as marker-assisted selection (MAS) and QC analysis require genotyping of a large number of samples with a small number of SNPs, which can more economically be achieved through uniplex (single-plex) genotyping platforms^23,27^. Currently, Kompetitive allele-specific PCR (KASP) is one of the most widely used uniplex genotyping platforms for applications requiring from one to a moderate number of markers, and offers reasonable cost, efficiency and flexibility^27,28^. KASP genotyping can be carried out in 96-, 384- and 1536‐well PCR plates and genotyping costs go down as number of wells go up^24^. Once SNPs of interest have been identified through the different approaches such as GBS and DArTseq, they must be converted for use as KASP assays and validated.

In rice, our team recently reported 339 diagnostic SNPs that can be used in correcting taxonomic misclassification of *O. barthii*, *O. glaberrima*, *O. longistaminata* and the two *O. sativa* subspecies^9,29^. These diagnostic markers require conversion for use in KASP assays and validation prior to use for quick and low-cost QC analysis. The present study presents the custom KASP assays created from the DArTseq-based *Oryza* species and subspecies diagnostic SNPs and the results of their validation. The aim was to recommend a smaller set of the best diagnostic SNPs for routine use in taxonomic curation of the four rice species and two subspecies conserved at different genebanks.

## Materials and methods

### SNP conversion to PCR-based markers (KASP conversion)

Based on the 332 DArTseq-based SNPs that were diagnostic for rice species/subspecies categorization reported in our previous study^5^ and seven newly identified DArT SNPs^9^, we submitted position and context sequence (the 50 base pairs (bp) on either side of the SNP) for 224 SNPs to the LGC Biosearch Technologies service laboratory, Hoddesdon, UK (https://www.biosearchtech.com/). Context sequence was found by BLASTing each 64-bp diagnostic DArTseq SNP sequence minus the common 5-bp adaptor/promoter against the *Oryza sativa* japonica reference genome in Gramene (http://www.gramene.org/). We copied 100–120-bp of sequence and marked the position of each SNP of the top BLAST hit for each of the 224 chosen DArTseq SNPs (Supplementary Table S1). LGC Biosearch Technologies service lab successfully designed KASP oligonucleotide assays for 158 of the 224 SNPs submitted.

### KASP marker testing and validation

The converted PCR-based markers appropriate for use in KASP assays were then used to genotype 80 DNA samples (Supplementary Table S2) representing *O. glaberrima* (18), *O. barthii* (18), *O. longistaminata* (9), *O. sativa* spp. *indica* (20) and *japonica* (15). All 158 KASP SNPs successfully produced genotype calls of which; (a) 20 SNPs were monomorphic across the 80 DNA samples; (b) 51 SNPs either generated too much missing data, too many heterozygotes, very low MAF and/or PIC, or were not taxonomically diagnostic; (c) 87 SNPs appeared to be diagnostic of which (d) 22 SNPs did not show a consistent pattern per species/subspecies (more details are provided in the results). After removing these 22, the remaining 65 were validated using 625 DNA samples representing *O. barthii* (88 samples), *O. glaberrima* (169), *O. longistaminata* (69), *O. sativa* spp. *indica* (178) and *O. sativa* spp. *japonica* (121) (Supplementary Table S2). All accessions used in this study were collected from the Rice Biodiversity Center for Africa (RBCA-AfricaRice) in Côte d’Ivoire. Details on the KASPar principle, amplification of targeted region, fluorescence detection and allele calling are available at https://biosearch-cdn.azureedge.net/assetsv6/KASP-genotyping-chemistry-User-guide.pdf. Cluster plots (Supplementary Figure S1) generated from the genotype data sets received from the LGC Biosearch Technologies service laboratory were visualized using SNPviewer2 v.4.0.0 (LGC Genomics, Teddington TW11 0LY, UK). Data were prepared for rapid navigation and comparisons between species and subspecies using TASSEL v.5.2.59^30^.

### Statistical analyses

Statistical values including Minor Allele Frequency (MAF) and heterozygous marker ratio for each SNP from the 80 genotypes were estimated using TASSEL v.5.2.59^30^. Fundamental measure of genetic diversity parameters such as Polymorphic information content (PIC) and gene diversity (GD) of a locus, also known as expected Heterozygosity (He) were computed using PowerMarker v3.25^31^. Based on 625 genotypes, a QC subset of SNP markers that were highly diagnostic for the four rice species (*O. barthii*, *O. glaberrima*, *O. longistaminata* and *O. sativa*) and the two subspecies (lowland *O. sativa *spp.* indica* and upland *O. sativa *spp. *Japonica*) was selected. The SNPs were selected by comparing the total number of accessions per species/subspecies genotyped and those that were successfully grouped by the markers used, focusing on the minor allele^32,33^. Because mismatches among positive controls genotyped in the same plate using KASP genotyping may account for up to 2.9%^23^ of the polymorphisms, we decided to select the best diagnostic markers which were SNPs that successfully clustered more than 97% of accessions per species/subspecies tested.

GenAlEx software version 6.503^34^ was used to perform the analysis of molecular variance (AMOVA) to detect the genetic variance within and among populations using the *PhiPT* value (analogue of F_ST_ fixation index) with 999 permutations. Each data set was converted to phylo “interleaved” format and imported in R v4.0.3^35^ using the *ape* package version 5.4-1^36^. Neighbor-joining (NJ) trees were built with a maximum likelihood approach and bootstrap analysis with 200 replicates using R/*phangorn* package version 2.5.5^37^. “Newick” format of Trees were exported and further refined using FigTree 1.4.3 program (http://tree.bio.ed.ac.uk/software/figtree/). We ran Principal Component Analysis (PCA) in TASSEL v.5.2.43. The first three principal components (PCs) were plotted for visual examination using R/*scatterplot3d* package version 0.3–41^38^ with species/ecotype as a categorical variable. The power of the selected diagnostic markers to correctly assign each individual to a distinct population was investigated performing a multivariate DAPC (Discriminant Analysis of Principal Components) method using R/*adegenet* package version 2.1.3^39,40^. For that analysis, three methods were used, as follows: (i) the number of clusters was assessed using the function *fnd.clusters* (assuming 20 as the maximum); (ii) the optimal number of clusters and/or groups in our data set were estimated using the Bayesian information criterion (BIC) and Silhouette Method using cluster package version 2.1.1^41^ in R; (iii) the DAPC result was presented as a barplot^42^.

### Ethical standards

Experimental research and field studies on plants (either cultivated or wild), including the collection of plant material, has complied with relevant institutional, national, and international guidelines and legislation. Seed samples were obtained from the AfricaRice genebank, Cote d'Ivoire. All accessions/genotypes used in this study are public goods and freely available for non-commercial purposes.

## Results

### Selection of a diagnostic SNP set

KASP primers for 158 of 224 sequences of DArTseq-based SNP species/subspecies diagnostic markers were converted and successfully produced genotype calls. This represented 70% of the 224 DArTseq-based SNP markers selected and mapped to the 12 rice chromosomes (Supplementary Table S1). Supplementary Table S3 and Supplementary Figure S2 summarizes the genetic diversity of the 158 KASP markers distributed in the 80 rice accessions. SNPs were filtered to remove SNPs that were monomorphic (MAF = 0%) or with MAF < 5%, PIC < 18%, heterozygous rates > 9%, and missing data > 24% (Supplementary Figure S2). This left 117 of the 158 SNPs. Genotype calls across the 80 accessions for these 117 SNPs identified 52 that were not taxonomically diagnostic or did not show a clear and consistent pattern, resulting in 65 KASP SNPs that appeared to be diagnostic. As described in Supplementary Table S4, six group-specific sets of SNPs were designed from the 65 KASPs markers obtained as follows: (i) SNPs that distinguish wild (*O. barthii*) and cultivated (*O. glaberrima*) African species (group 1, 4 SNPs); (ii) SNPs that distinguish complex (*O. barthii*/*O. glaberrima*) and complex (*O. sativa* and *O. longistaminata*) (group 2, 28 SNPs); (iii) SNPs that distinguish Asian species *O. sativa* from indigenous African species complex (*O. barthii*/*O. glaberrima*/*O. longistaminata*) (group 3, 16 SNPs); (iv) SNPs that distinguish *O. longistaminata* and complex (*O. barthii*/*O. glaberrima* and *O. sativa*) (group 4, 9 SNPs); (v) SNPs that distinguish lowland *O. sativa* spp. *indica* and complex (upland *O. sativa* spp. *Japonica*/*O. barthii*/*O. glaberrima*/*O. longistaminata*) (group 5, 7 SNPs)..

**Figure 1:**
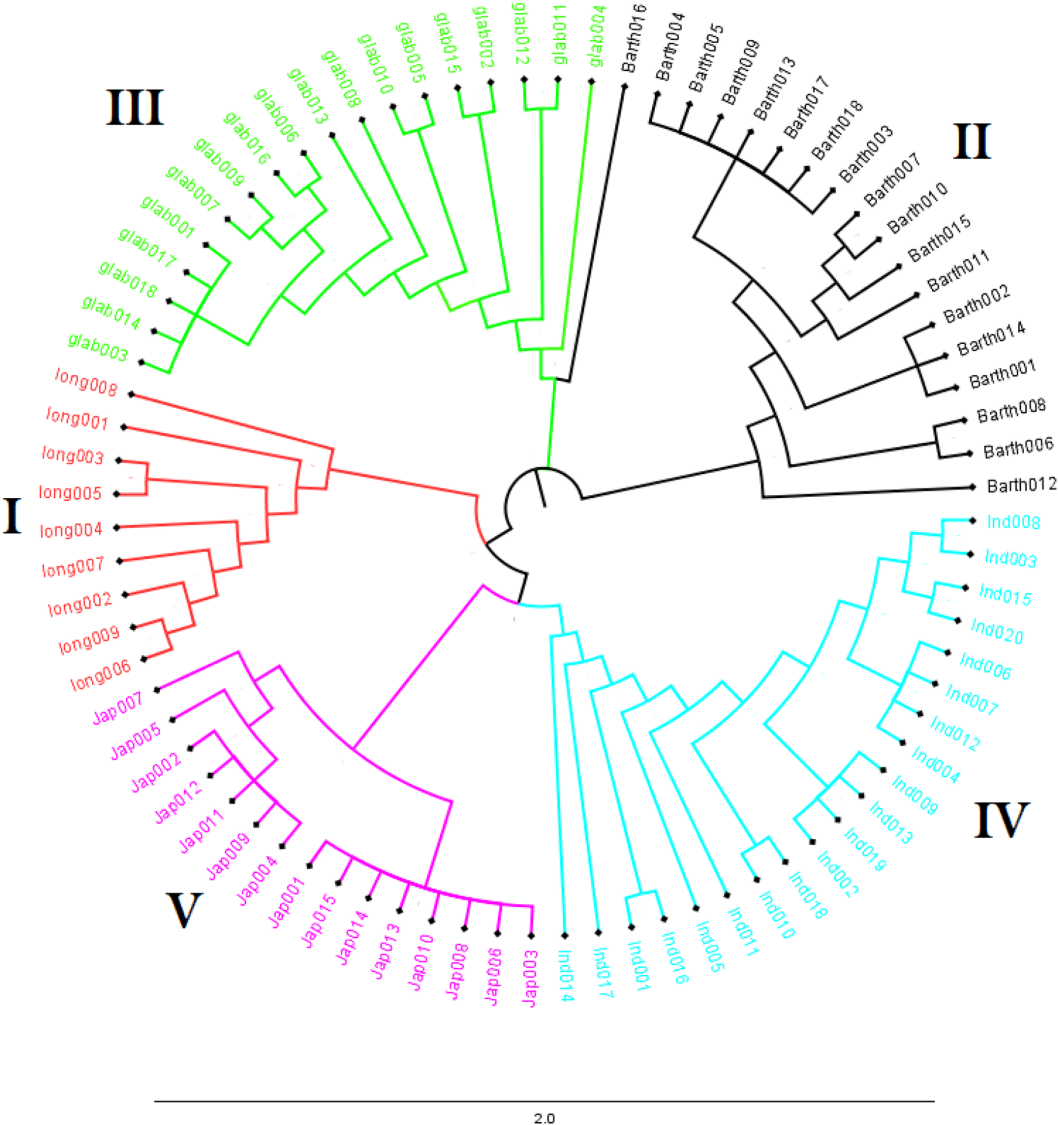
Phylogenetic tree constructed using the Neighbor-Joining method based on 80 rice accessions representing *O. glaberrima* (18), *O. barthii* (18), *O. longistaminata* (9), *O. sativa* spp. indica (20) and *O. sativa* spp. *japonica* (15) genotyped with 65 KASP SNPs diagnostic markers. Details about the 80 rice accessions are provided in the Supplementary Table S2. The colors in the tree correspond to subpopulations, as follows: Cluster **I** in red represents *O. longistaminata* accessions; Cluster **II** in black represents *O. barthii* accessions; Cluster **III** in green represents African rice *O. glaberrima* accessions; Cluster **IV** in blue represents Asian rice *O. sativa* spp. *indica* accessions; and Cluster **V** in pink represents Asian rice *O. sativa* spp. *japonica.*

Use of the 65 diagnostic KASP markers to seek subpopulations within the 80 rice accessions using PCA and NJ phylogenetic tree demonstrated their suitability to properly classify all accessions in this group. Three first three principal components accounted for 73%, 13% and 6% of the total molecular variance in the genotype data (cumulative 92%; Supplementary Figure S3). A plot of each of these three PCA showed five groups in the same way as the NJ phylogenetic tree (Supplementary Figure S3 and Fig. 1). The eighty rice accessions were clustered into the expected five groups as follows: (a) cluster **I** contained 9 accessions of the wild rice species *O. longistaminata*; (b) the cluster **II** contained the 18 accessions of *O. barthii*; (c) cluster **III** contained the 18 accessions of *O. glaberrima*; (d) cluster **IV** contained the 20 accessions of Asian rice*, O. sativa* spp. *indica* groups adapted to the lowland and finally (e) the cluster **V** contained 15 accessions of the Asian rice, *O. sativa* spp *japonica* to the upland ecologies. For routine QC and taxonomic classification of genebank materials, however, 65 markers is considered too many to run.

### Validation of a diagnostic SNP subset

**Figure 2:**
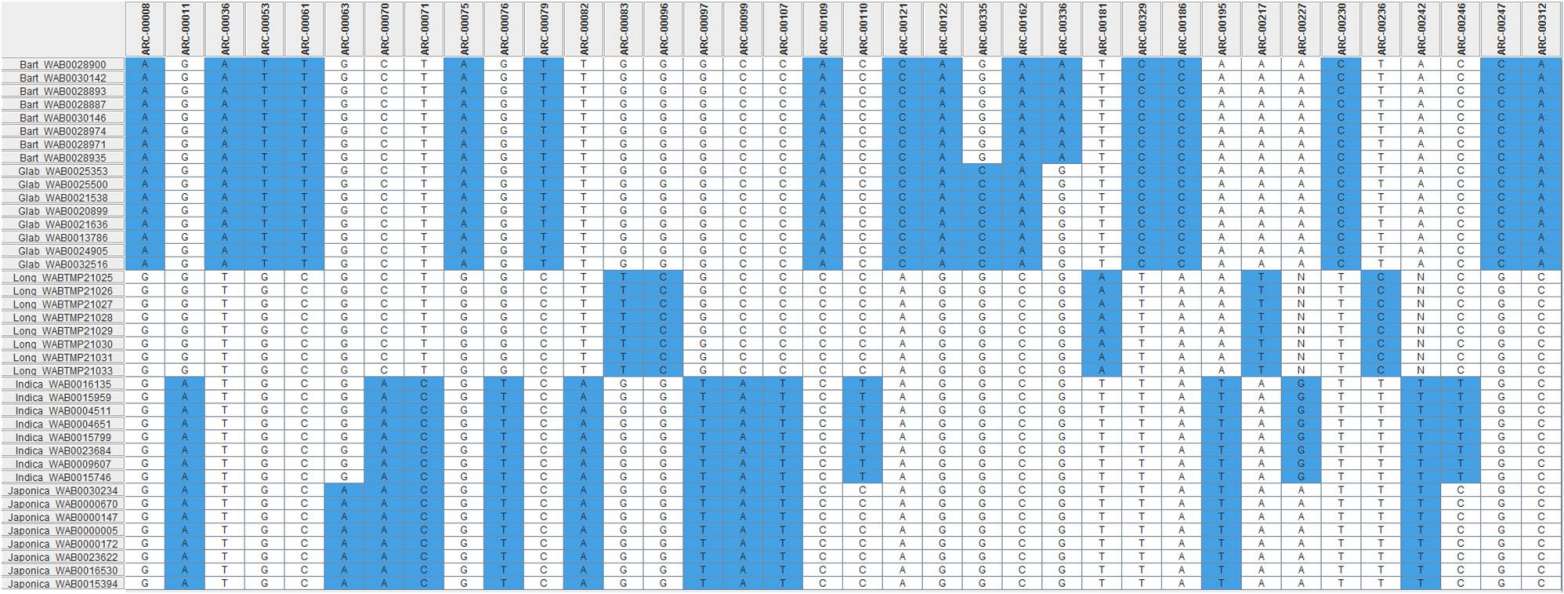
Haplotype pattern of 36 diagnostic SNP markers recommended for “broad” quality control genotyping between *O. barthii* and *O. glaberrima* (2 SNPs), between the two African species *O. barthii*/*O. glaberrima* and the complex *O. sativa* and *O. longistaminata* (16 SNPs), between the Asian rice *O. sativa* and the three African species *O. barthii*/*O. glaberrima*/*O. longistaminata* (9 SNPs)*,* between *O. longistaminata* and O. *barthii/O. glaberrima* and *O. sativa* (5 SNPs)*,* between lowland *O. sativa* spp*. indica* and the complex Upland *O. sativa* spp*. japonica/O. barthii/O. glaberrima/O. longistaminata* (3 SNPs), and between Upland *O. sativa* spp*. japonica* and the complex lowland *O. sativa* spp*. indica/O. barthii/O. glaberrima/O. longistaminata* (1 SNPs). Each species or ecotype is represented by eight randomly selected accessions, which are listed in rows on the left side with prefix (Bart *O. barthii*, Glab *O. glaberrima*, Long *O. longistaminata*, Indica *O. sativa* spp. *indica*, *japonica* O. sativa spp. *japonica*), followed by accession number. The SNP IDs are listed as column headings. Each column has two alleles that are shaded either in white or blue. See Supplementary Table S2 for details on accessions, Supplementary Table S1 for SNP summary.

To develop a more cost-effective routine QC genotyping approach, we searched for a smaller number of markers able to efficiently differentiate the six specific taxa. The quality of the 65 KASP markers from 625 rice accessions is shown in Supplementary Table S4, which summarizes, for each marker, the percentage of each species/subspecies correctly grouped based on each marker. We selected all SNPs that correctly identified at least 97% of accessions with their taxonomic group; 36 of the 65 diagnostic SNPs were thus selected. Of these, 16 could identify accessions belonging to group 2; nine of the 16 SNPs could assign accessions to group 3; five could assign accessions to group 4; three could assign accessions to group 5, and the one SNP identified previously could still assign accessions to group 6 (Supplementary Table S4). While no diagnostic SNP marker was found for the species in group 1 according to this first filtering method adopted, two SNPs (ARC-00335 and ARC-00336) working together could assign accessions to group 1. The 36 selected SNPs are located on all rice chromosomes except 1, 4, and 11 (Supplementary Table S1). We also present in Fig. 2 the haplotype pattern of 36 diagnostic KASP SNP markers across the four AA genome *Oryza* species and the two subspecies studied. Information on the 36 diagnostic SNPs markers in the 625 rice accessions matched the information on the original DArT markers, and are summarized in Supplementary Table S5 and Supplementary Figure S5.

**Figure 3:**
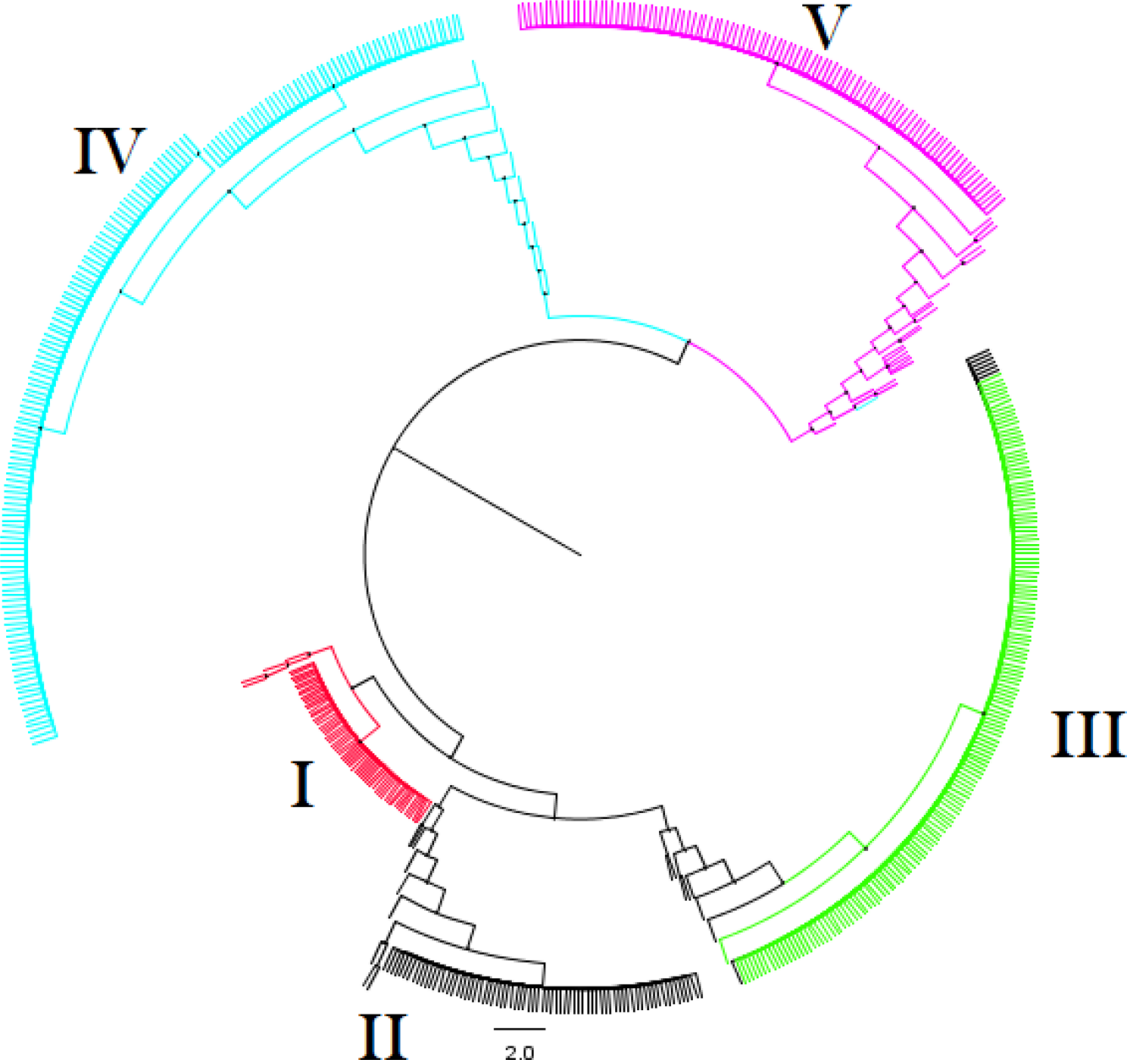
The phylogenetic tree constructed using the Neighbor-Joining method based on 625 rice accessions representing *O. barthii* (88 samples), *O. glaberrima* (169), *O. longistaminata* (69), *O. sativa* spp. indica (178) and *O. sativa* spp. *japonica* (121), plotted with 36 KASP SNPs diagnostic markers. Details about the 625 rice accessions were provided in the Supplementary Table S2. The colors in the tree correspond to subpopulations. Cluster **I** in red represents *O. longistaminata* accessions, cluster **II in** black represents *O. barthii* accessions, cluster **III** in green represents African rice *O. glaberrima* accessions, cluster **IV** in blue represents Asian rice *O. sativa* spp *indica* accessions, and cluster **V** in pink represents Asian rice *O. sativa* spp *japonica.*

Genetic variance partitioning through the AMOVA test based on *PhiPT* values indicated that most of the genetic diversity occurred among species/subspecies (97%), with within species diversity accounting for only 3% of the observed genetic diversity (Table 1). The *PhiPT* value for 625 rice accessions was 0.974 (P < 0.001) (Table 2). Pairwise *PhiPT* genetic distances for all populations were significantly greater than 0 (P < 0.001) and explained discernible population differentiation, ranging from 0.81 to 0.99 between different groups (Table 2). To investigate relationships among populations, a NJ phylogenetic tree was plotted using the 36 diagnostic markers across the 80 and 625 accessions (Supplementary Figure S4 and Fig. 3), resulting in five groups: (a) cluster **I** with accessions of the wild rice species, *O. longistaminata*; (b) cluster **II** that contained accessions of the wild rice species, *O. barthii*; (c) cluster **III** that contained accessions of African rice, *O. glaberrima*; (d) cluster **IV** that contained accessions of Asian rice*, O. sativa* spp. *indica* groups adapted to the lowland and finally; (e) cluster **V** that represented Asian rice, *O. sativa* spp. *japonica* to the upland ecologies. As indicated in Fig. 3, seven *O. barthii* accessions were grouped with African rice (*O. glaberrima*) in cluster III.

**Table 1:** Analysis of molecular variance (AMOVA) of 625 rice accessions based on the 36 KASP markers.

Source of variation	df	SS	MS	Est. var.	%	P value
Among pops	4	4848.905	1212.226	9.992	97	< 0.001
Within pops	620	162.642	0.262	0.262	3	< 0.001
Total	624	5011.547		10.254	100	< 0.001

*df* degree of freedom, *SS* sum of squares, *MS* mean squares, *Est. var.* estimate of variance, *%* percentage of total variation, P-value is based on 999 permutations.

**Table 2:** Pairwise differentiation (*PhiPT*) among four rice species and two subspecies based on 36 KASP markers.

	*O. longistaminata*	*O. barthii*	*O. glaberrima*	*O. sativa *spp.* japonica*	*O. sativa *spp*. indica*
*O. longistaminata*		0.001	0.001	0.001	0.001
*O. barthii*	0.976		0.001	0.001	0.001
*O. glaberrima*	0.988	0.811		0.001	0.001
*O. sativa *spp*. japonica*	0.971	0.978	0.987		0.001
*O. sativa *spp*. indica*	0.964	0.975	0.983	0.862	

PhiPT values are shown below the diagonal. Probability was based on 999 permutations, shown above the diagonal.

**Figure 4:**
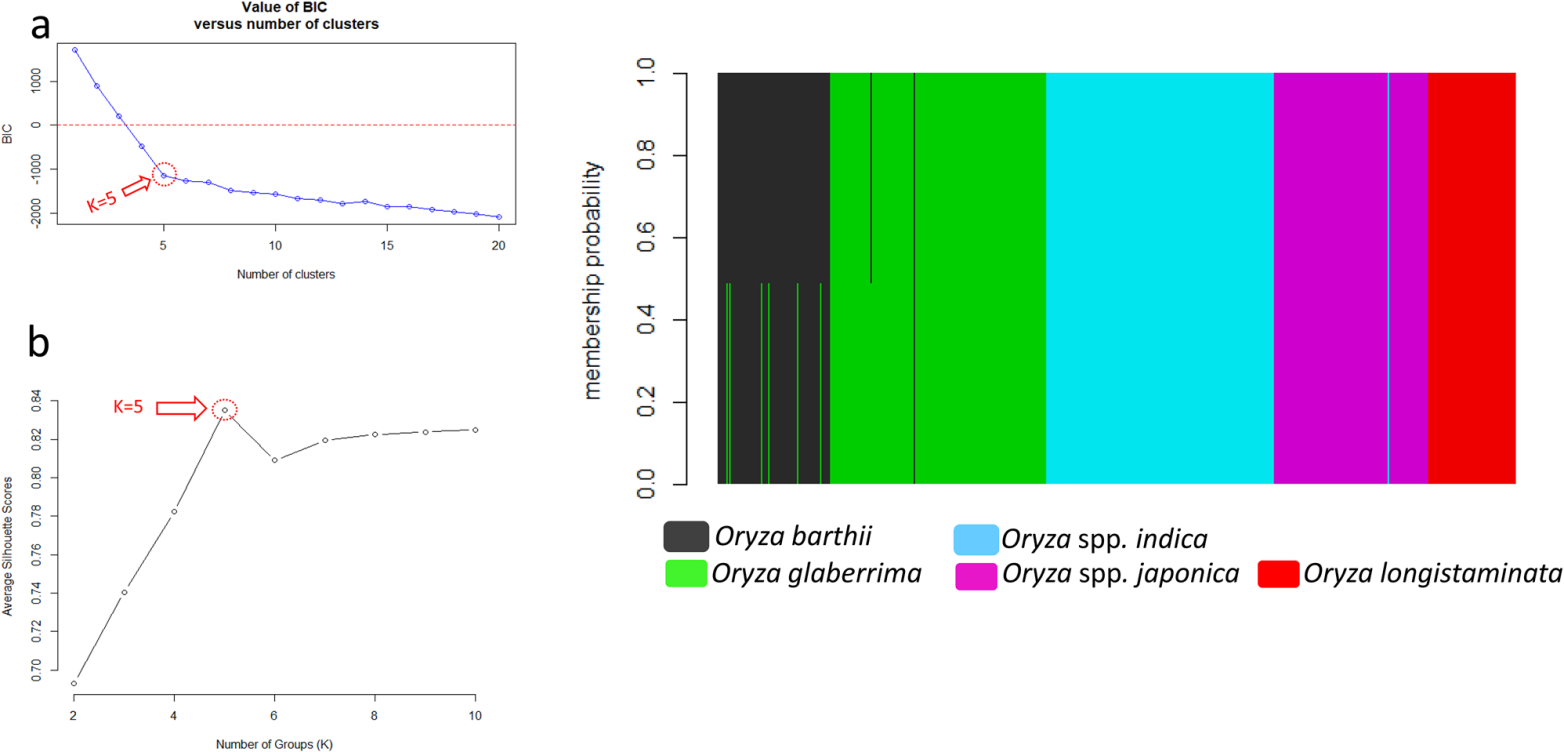
DAPC scatterplots based on the 36 diagnostic KASP markers. (**a**) K number selected based on Bayesian information criterion (BIC) value for cluster up to K = 20; (**b**) Silhouette score by number of groups (K); and (**c**) DAPC plot for the optimal number of clusters, K = 5. Each bar in (**c**) represents an individual (accession) that is coloured according to its probability of belonging to a given cluster.

The first three principal components of the 36 diagnostic KASP SNPs genotyped in 625 accessions accounted for 76.8% to 99% of the molecular variation with eigenvalues from 5.9 to 0.01. The first, second and third axis explained 77%, 14% and 6% of the overall variance, respectively (Supplementary Figure S6). The scatter plot of first three PCs revealed five groups in which three were very distinctive (Supplementary Figure S6). As observed with the N-J tree (Fig. 3), some accessions from *O. barthii* species were very close to *O. glaberrima* species in the PCA plot (cluster II and III). Genetic distinctness of these rice species/subspecies was evaluated with the 36 diagnostic markers using DAPC. Bayesian information criterion (BIC) and Silhouette score value revealed K = 5 as the optimal number of clusters, consistent with partitioned variance between these groups of accessions (Fig. 4).

## Discussion

### KASP marker development by DArT SNP sequence

Genebank managers are tasked with ensuring the accurate identification of species and maintenance of genetic integrity of the accessions in their ex situ collections by, as much as possible, preventing human errors during routine genebank operations. These errors are mainly caused by mislabelling and physical as well as genetic admixture during various gene banking activities. Many genebanks do not have the resources and capacity to assess their collections in order to verify the correctness of their passport (origin) data and to undertake correct species identification^5,21^. Dense genotypic information can be used for better understanding of genetic diversity, enable selection of accessions possessing selected traits, and to facilitate gene identification and use of plant genetic resources. While the same data can serve as molecular passport data to complement, corroborate and correct traditional passport records^43^, QC genotyping based on markers is less expensive and faster than phenotypic evaluation^5^. However, fewer markers and cheaper genotyping must be available for QC genotyping compared to the dense genotypic information genotyping, as recommended by IRRI (http://gsl.irri.org/genotyping/qualitycontrol-panel/indica-rice-qc-10-snp-panel)^5^ and the International Maize and Wheat Improvement Center (CIMMYT)^44^.

KASPar or KASP is a user-friendly SNP platform that is cost efficient for smaller numbers of markers (< 200)^45^, which is what is needed for marker-assisted recurrent selection and marker-assisted backcrossing^46^, and routine QC analysis. QC analysis can use up to a few dozen markers, depending on the range and partitioning of variation in a group of taxa^23^. In our study, species discriminating KASP markers were developed for four rice species (*O. barthii*; *O. glaberrima*; *O. longistaminata* and *O. sativa*) and two subspecies (lowland *O. sativa *spp. *indica* and upland *O. sativa *spp.* japonica*), offering a new tool for routine QC genotyping of these rice species for conservation in genebanks. As we demonstrated in our previous study, no DArT SNPs from the 332 diagnostic SNPs selected could differentiate *O. barthii* from *O. glaberrima* accessions^29^. The present study, however, permitted us to select 7 new SNPs that could be used to distinguish them^9^. This was possible because of the larger number of markers used in the present study compared to the previous one (46,818 vs 31,739 SNPs, respectively). Not all SNP sequences can be converted to KASP assays, as nearby polymorphisms and high GC content in the sequence around the SNP hinder primers from proper annealing and thus stop assays from proper amplification. In this study, KASP markers were successfully developed for 158 of 224 DArTseq-based SNPs sites submitted for conversion (Supplementary Table S1)**.** This constitutes a design success rate of 71%, similar to what has been reported previously in other crops^47^. Failure to successfully convert some of the markers can possibly be due to nearby polymorphisms in the sequences of the accessions included in this panel or the presence of paralogous sequences. Using optimized PCR conditions, it might have perhaps been possible to improve the conversion rate in some of the failed assays. Nevertheless, these efforts generated more than sufficient KASP markers to differentiate all taxa in the study.

As suggested by Semagn and colleagues^23^, we chose KASP assays that had a low missing data rate, low heterogeneity, and very high (97%) scoring success rate in our panels, which contained a high number of lines to ensure diversity within the taxa were represented. This appeared to be a good approach to validate diagnostic KASP markers, and has been followed by others as well^44^. Most of the KASP markers selected for broad quality control in the present study generated less than 11% missing values, except one marker (ARC-00336 = 21% missing data) (Supplementary Table S5), but was kept because it is highly diagnostic for differentiating between *Oryza barthii* and *Oryza glaberrima.* This marker should not be a problem, because it produced more missing data with *Oryza sativa* accessions adapted to lowland ecologies, which are amply differentiated by other KASP assays. Heterogeneity ranged between 0 to 1% across the 625 samples used for validation testing (Supplementary Table S5).

### Genetic diversity and differentiation

Two diversity indices were estimated using both DArT and KASP SNPs markers: (i) the Polymorphism Information Content (PIC) value, which is often used to measure the informativeness of a genetic marker for linkage studies^48^ and (ii) Minor allele frequency (MAF), which helps to differentiate the common and rare SNPs in the population^49^. As presented in the Supplementary Table S3, genetic diversity analysis was performed to compare the ability of DArT and KASP markers to capture genetic variance. The PIC and MAF average values computed in the entire group of markers converted to KASP (158 SNPs) were higher than those previously found with the original set of DArT markers used for conversion (0.32 and 0.24 versus 0.23 and 0.17, respectively). These small differences could be due to the very large numbers of accessions used in our previous study or the large numbers of DArT markers, most of which did not have high PIC or MAF values. The present KASP markers presented consistent clustering patterns as presented via PCA, cluster analysis, and population structure analysis across the 625 accessions.

AMOVA of the 625 accessions genotyped with the 36 KASP markers indicated much higher genetic diversity between populations than within (Table 1), which has also been reported in previous studies on rice^50^, and is a good indicator that the species are indeed distinct, and that these KASP markers can properly measure the differentiation. Also, one reason the genetic diversity was much higher between populations vs within populations is attributable to the ascertainment bias of the set of 36 SNPs, which had been specifically selected to have higher polymorphism between populations. The *PhiPT* calculations provide an estimation of the structure of genetic variation among populations, groups or species, with < 0.05 indicating little, 0.05–0.15 moderate, 0.15–0.25 great, and > 0.25 very great genetic differentiation^51^. All pairwise population *PhiPT* values in this study were high for the species/subspecies compared, and ranged from 0.811 (*O. barthii* vs *O. glaberrima*) to 0.988 (*O. glaberrima* vs *O. longistaminata*) (Table 1). As expected, these values are higher than reported in recent studies on genetic differentiation within *Oryza sativa* L.^52,53^ but similar to between-species differentiation reported previously^9,54^. Together, the results reported here and in previous studies indicate that KASP markers are highly effective for differentiation of the four rice species and two sativa subspecies under study.

### Genetic relationship and population distinctness

In germplasm characterization, phylogenetic trees are primarily used to define groups, and measure the divergence between them. This can be useful for understanding the broader pattern of phylogenetic and evolutionary relationships, and more practically, to identify potential duplicates and to select a subset of accessions that capture the genetic variation of a given group^55^. Both PCA and cluster analysis provided similar illustrations of the relationships in the rice germplasm studied here; the methods can be combined into DAPC, an approach that used PCA for data transformation prior to a discriminant analysis (DA), which then partitions genetic variation to maximize differences between clusters while minimizing within-cluster variation^40,42^. We used this method to access the ability of the diagnostic markers to correctly identify clusters of individuals known to be evolutionarily related.

As shown in Figs. 3, 4 and Supplementary Figure S3, all accessions were separated into five groups, as per the traditionally and taxonomically accepted classification. The *O. longistaminata* species is morphologically distinct from all other AA-genome species in *Oryza,* and here, all grouped tightly together into cluster I in the PCAs and N-J trees. As expected, *O. barthii* and *O. glaberrima* (clusters II and III, respectively) are very similar genetically^56^ and also have a close evolutionary relationship, as the African wild species *O. barthii* is the direct and recent ancestor of cultivated *O. glaberrimai*^57–59^ (Supplementary Figures S3 and S4). Cluster II contains 81 of the 88 *O. barthii* accessions, which were differentiated from other species/subspecies based on the 2 KASP diagnostic markers (ARC-00335 and ARC-00336) (Fig. 3).These two markers revealed contrasting haplotypes between 100% of the *O. glaberrima* and 92% of the *O. barthii* accessions genotyped (Figs. 2 and 3).

The remaining seven accessions of *O. barthii* (WAB0038210, WAB0038227, WAB0009247, WAB0028947, WAB0028983, WAB0038213 and WAB0038205) clustered with the African cultivated rice *O. glaberrima* accessions (see black points cluster III; Fig. 3), and showed the distinctive haplotype of marker ARC-0033 (referenced as the major allele “G” instead of the desirable minor allele “A”) of *O. glaberrima*. Accordingly, we suspect that these seven accessions could be intermediate plants or “weedy” accessions, which result from hybridization between *O. barthii* and *O. glaberrima*. Hybridization happens easily given that these two species are inter-fertile, and has been reported previously^22,60^. As presented on Supplementary Figure S2, one japonica accession clustered half way between groups 4 and 5. This may be an admixture between indica and japonica, which has also been previously reported in several studies^33,61,62^. The phylogenetic analyses provide additional confirmation that the KASP markers have sufficient capacity to correctly classify genetically diverse rice accessions.

### Diagnostic KASP markers for classification of rice germplasm

Previous studies^23,63,64^ recommended two or three SNPs per chromosome and a minimum of 20–25 KASP markers to serve as diagnostic markers to distinguish genotypes in germplasm collections; we have worked to follow these guidelines. However, only one SNP per chromosome was retained for each of the group-specific G1, G4, G5 and G6. We also retained 16 SNPs for group-specific G2. This number was slightly similar to that suggested in our previous study for group-specific germplasm category (11 to 14 SNPs)^5^ and was higher than that found as diagnostic for the remaining group-specific categories of germplasm (between 1 to 9 SNPs). However, they allowed the correct discrimination of the four species and two subspecies considered in this study via their haplotype pattern, PCA, cluster analysis, and population structure analysis. To increase this small number of diagnostic SNPs specific per germplasm group, we suggest that more SNPs can be converted from DArT to KASP assay. However, for all other groups, fewer SNPs may be sufficient; in fact, a subset of 24 out of the 36 KASP SNP markers yielded the same results (Supplementary Figure S7) as analysis done using the entire set of 36 markers (Fig. 3) suggesting that this reduced number of markers may be sufficient, as long as they are the correct SNPs (and will distinguish all species/sub-species).

## Conclusions

In the present study we successfully developed robust and user-friendly, routine KASP genotyping assays for rice species conserved in various genebanks including the AfricaRice genebank. The KASP assays are based on previous DarTseq SNPs markers selected as diagnostic between various *Oryza* species. The assays are available at LGC genomics. The cost of KASP genotyping services is reasonable for CGIAR Centers and national agricultural research systems (NARS) in developing countries, which have agreements with KASP genotyping service providers. The cost per sample is reduced with higher numbers of data points generated, and service includes both DNA extraction and SNP genotyping^5,63^. The use of this service will help the AfricaRice genebank team and other genebanks to correct/update traditional passport data of their germplasm collections focusing on the four species (*O. barthii*, *O. glaberrima*, *O. longistaminata*, and *O. sativa* spp. *Indica* and *japonica*), and to identify and track cases of misclassification/mislabeling and physical germplasm contamination during repetitive genebank operations. We propose that subsets of 24 and 36 KASP-SNPs markers be employed for “rapid” and “broad” diagnostic activities, respectively, for rice germplasm. These markers constitute an important genomic resource to support the conservation and use of rice genetic resources.

## Supplementary Information

Supplementary Figures.

Supplementary Table S1.

Supplementary Table S2.

Supplementary Table S3.

Supplementary Table S4.

Supplementary Table S5.

## Data Availability

All relevant files are included in this article and its supplementary files. The raw genotype data of all accessions will be deposited in the public database upon acceptance of the manuscript.
