# Supplementary material for: Development and validation of diagnostic SNP markers for quality control genotyping in a collection of four rice (Oryza) species
Source: Sci Rep. 2021 Sep 20;11:18617. doi: 10.1038/s41598-021-97689-3 (PMC8452751; doi:10.1038/s41598-021-97689-3)
Supplement: Supplementary file 1 — Supplementary Figures. [file 41598_2021_97689_MOESM1_ESM.pdf]

Supplementary Figures

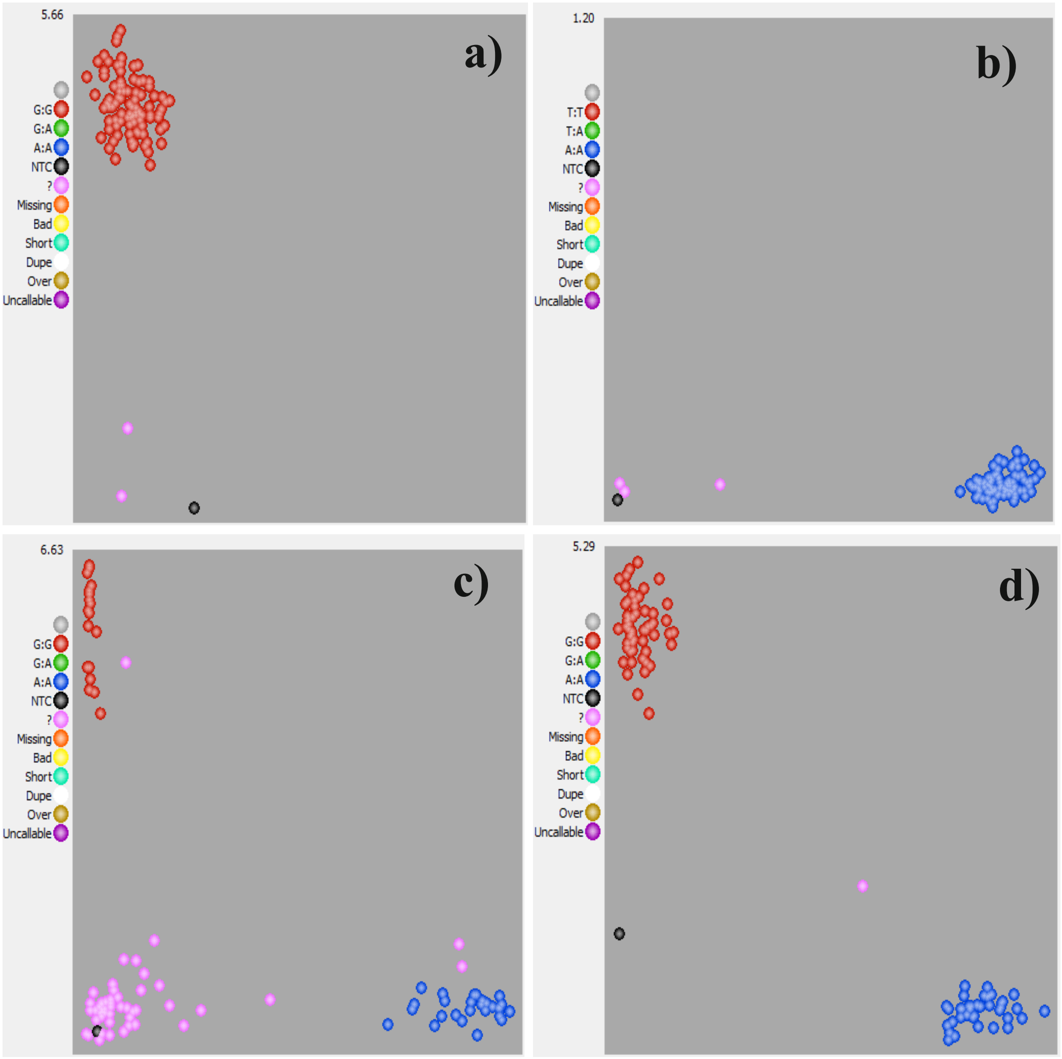

Supplementary Figure S1

Valid and invalid markers: (**a**) & (**b**) Monomorphic or invalid markers generates one fluorescent signal (**c**) invalid markers were not well amplified; (**d**) Polymorphic or valid markers amplified well. Red dots are homozygous for one allele, blue dots are homozygous for a second allele, black dots are negatives controls, pink dots are samples with no signal.

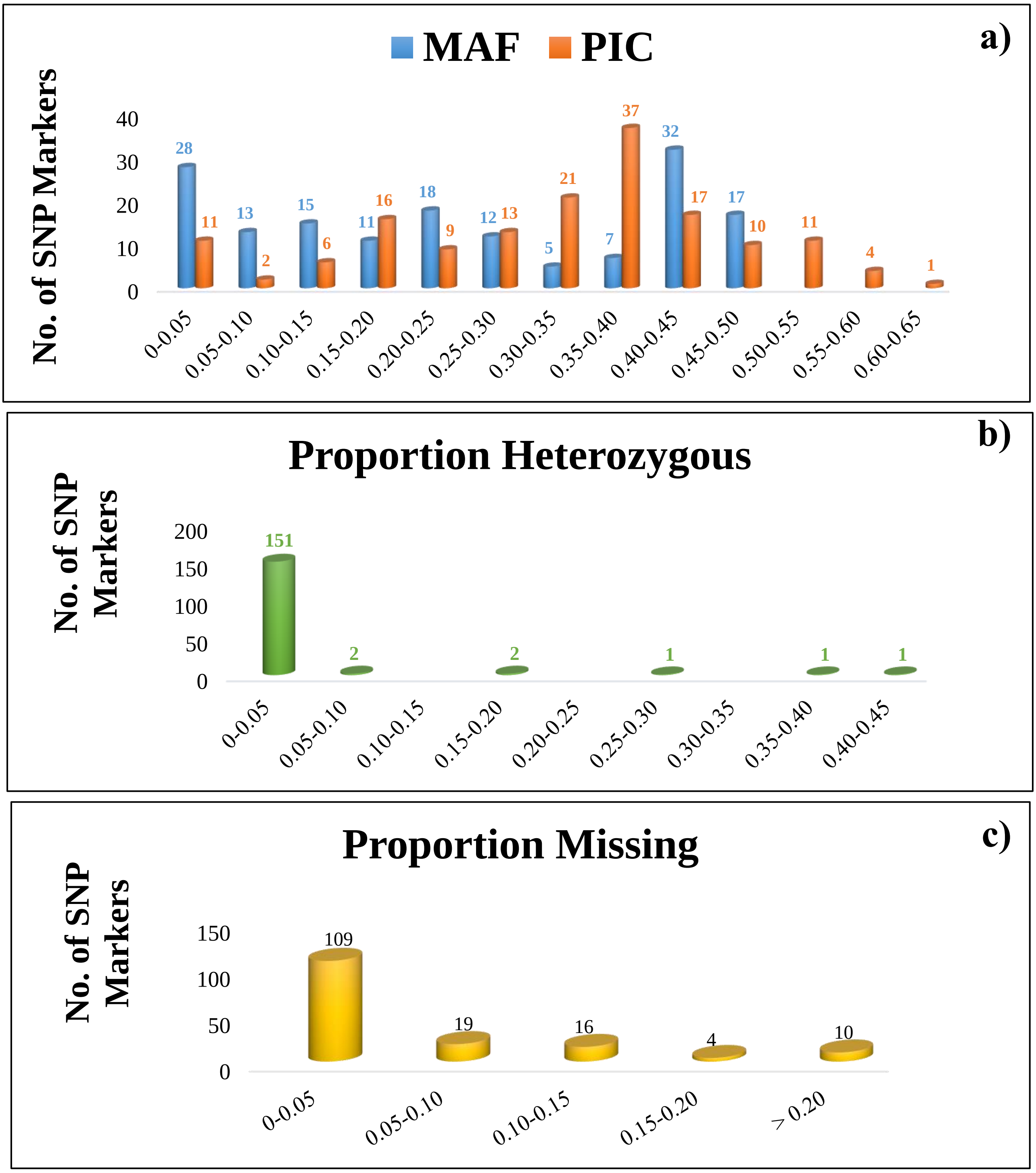

Supplementary Figure S2

Distribution of genetic diversity for 158 KASP-SNP markers in the 80 rice accessions. **(a)** Minor Allele Frequency (MAF) and polymorphic information content (PIC); **(b)** Proportion of Heterozygous; **(c)** proportion of missing data.

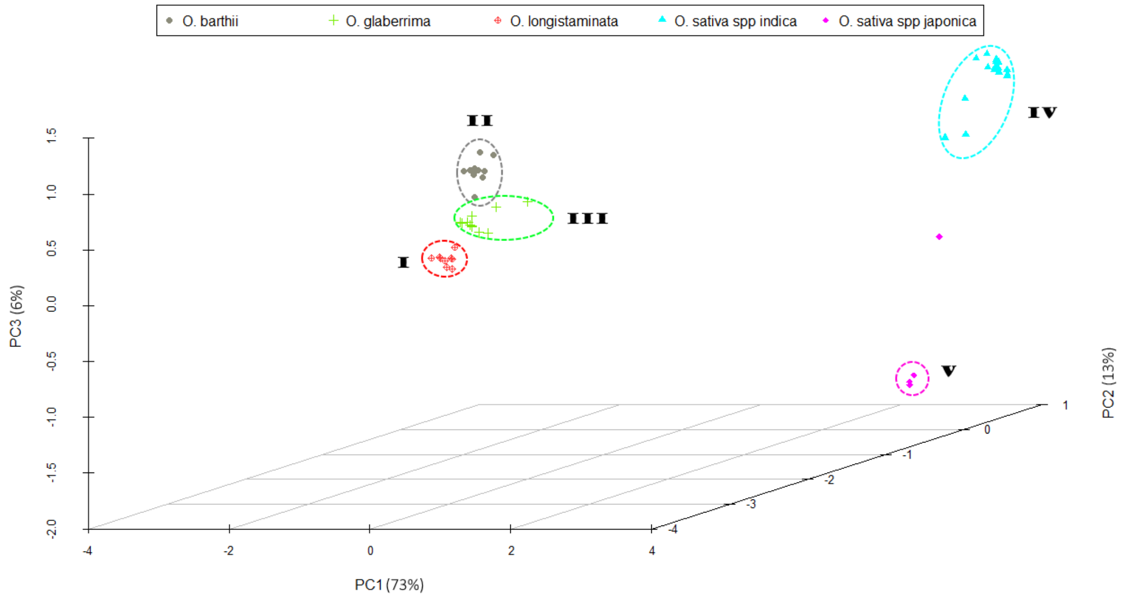

Supplementary Figure S3

Summary of the PCs from principal component analyses of 80 accession samples from *O. glaberrima* (18), *O. barthii* (18), *O. longistaminata* (9), *O. sativa* spp. *indica* (20) and *japonica* (15) based on 65 KASP-SNPs markers.

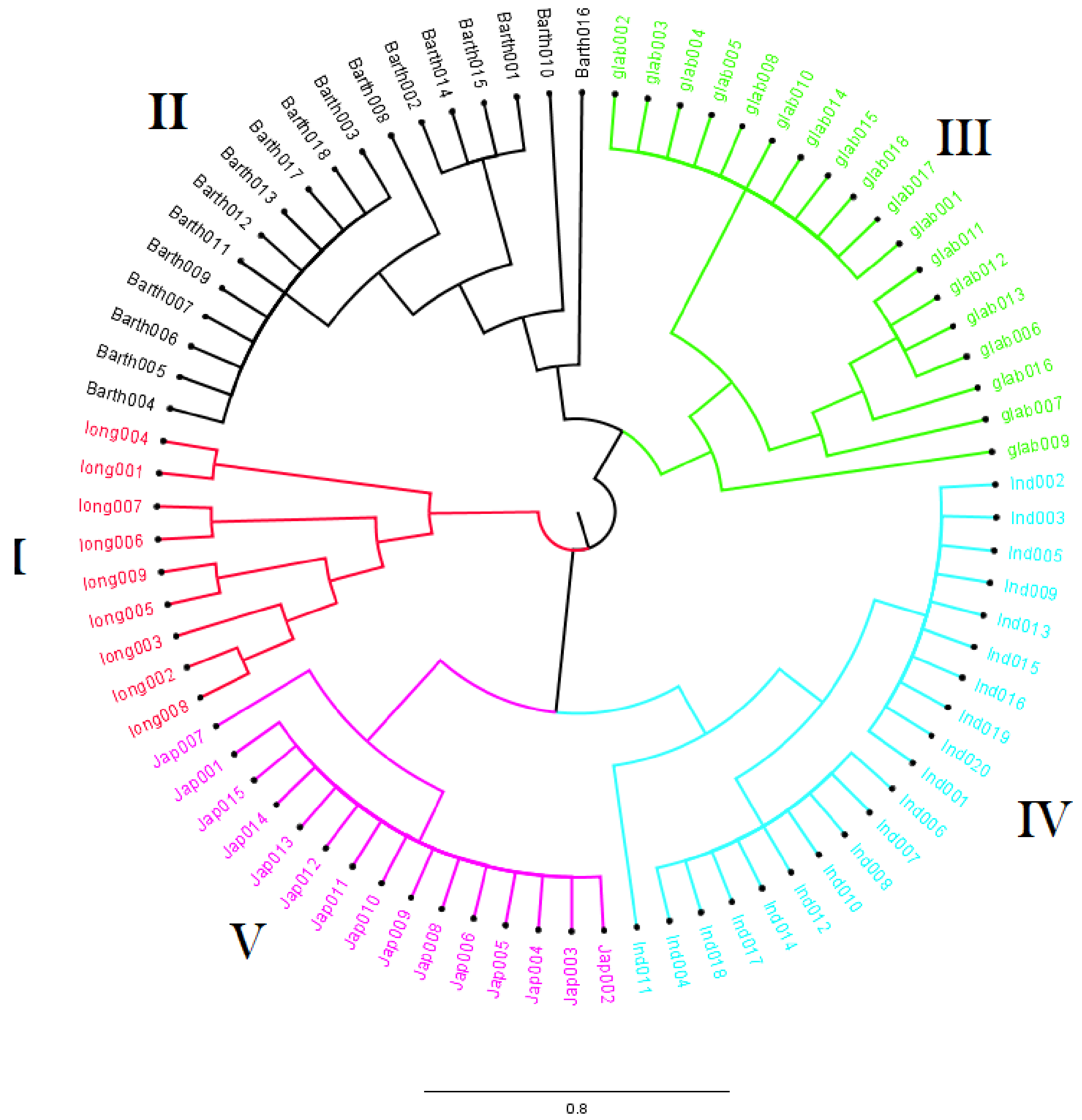

Supplementary Figure S4

Phylogenetic tree constructed using the Neighbor-Joining method based on 80 rice accessions representing *O. glaberrima* (18), *O. barthii* (18), *O. longistaminata* (9), O. sativa spp. indica (20) and *O. sativa* spp japonica (15) genotyped with 36 KASP SNPs diagnostic markers. Details about the 80 rice accessions are provided in the Supplementary Table S2. The colors in the tree correspond to subpopulations. Cluster I in red represents *O. longistaminata* accessions; Cluster II in black represents *O. barthii* accessions; Cluster III in green represents African rice *O. glaberrima* accessions; Cluster IV in blue represents Asian rice *O. sativa* spp indica accessions; and Cluster V in pink represents Asian rice *O. sativa* spp japonica.

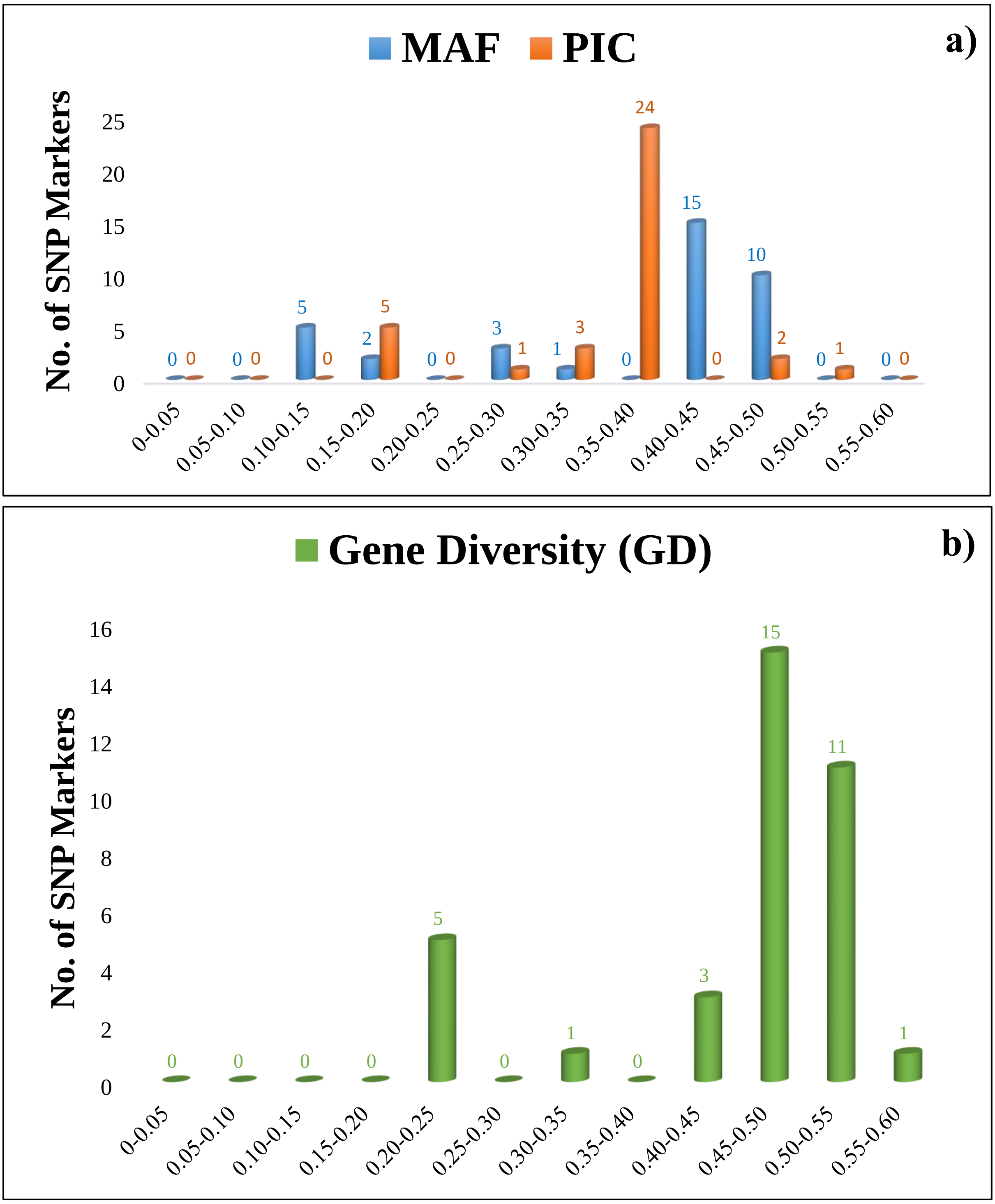

Supplementary Figure S5

Distribution of genetic diversity for 36 KASP-SNP markers in the 625 rice accessions. **(a)** Minor Allele Frequency (MAF) and polymorphic information content (PIC); **(b)** Gene diversity (GD) or expected heterozygosity (He).

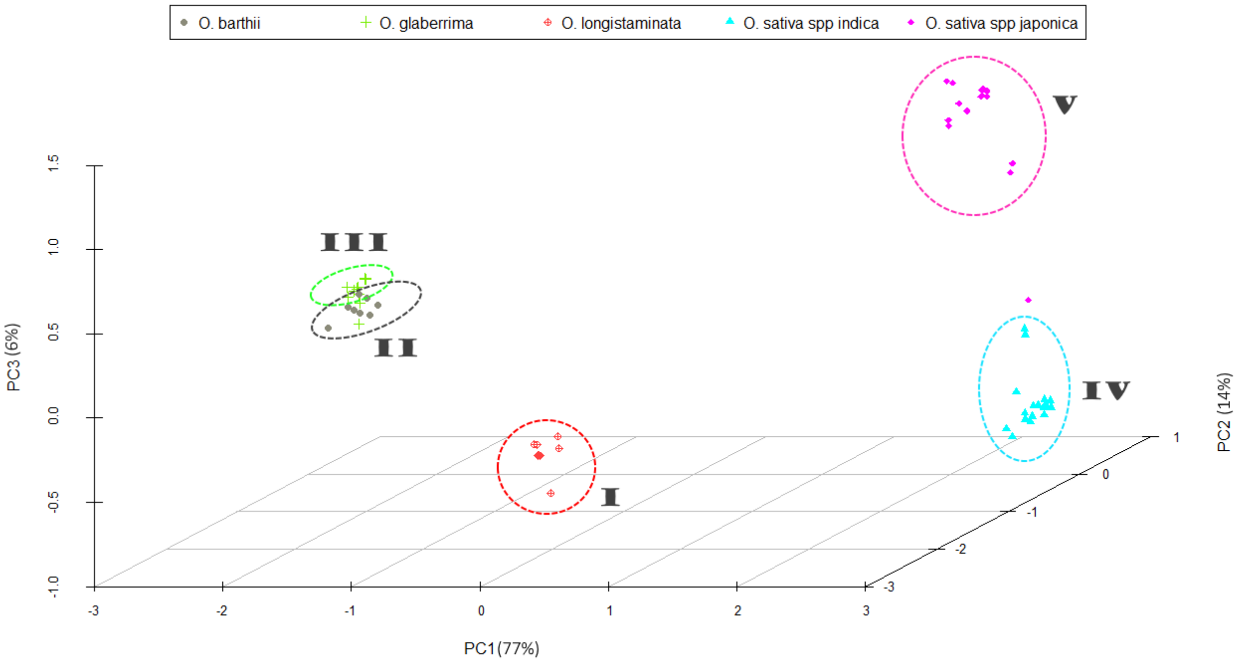

Supplementary Figure S6

Summary of the PCs from principal component analyses of 625 accessions samples from *O. barthii* (88 samples), *O. glaberrima* (169), *O. longistaminata* (69), *O. sativa* spp. indica (178) and *O. sativa* spp. japonica (121) based on 36 KASP-SNPs markers.

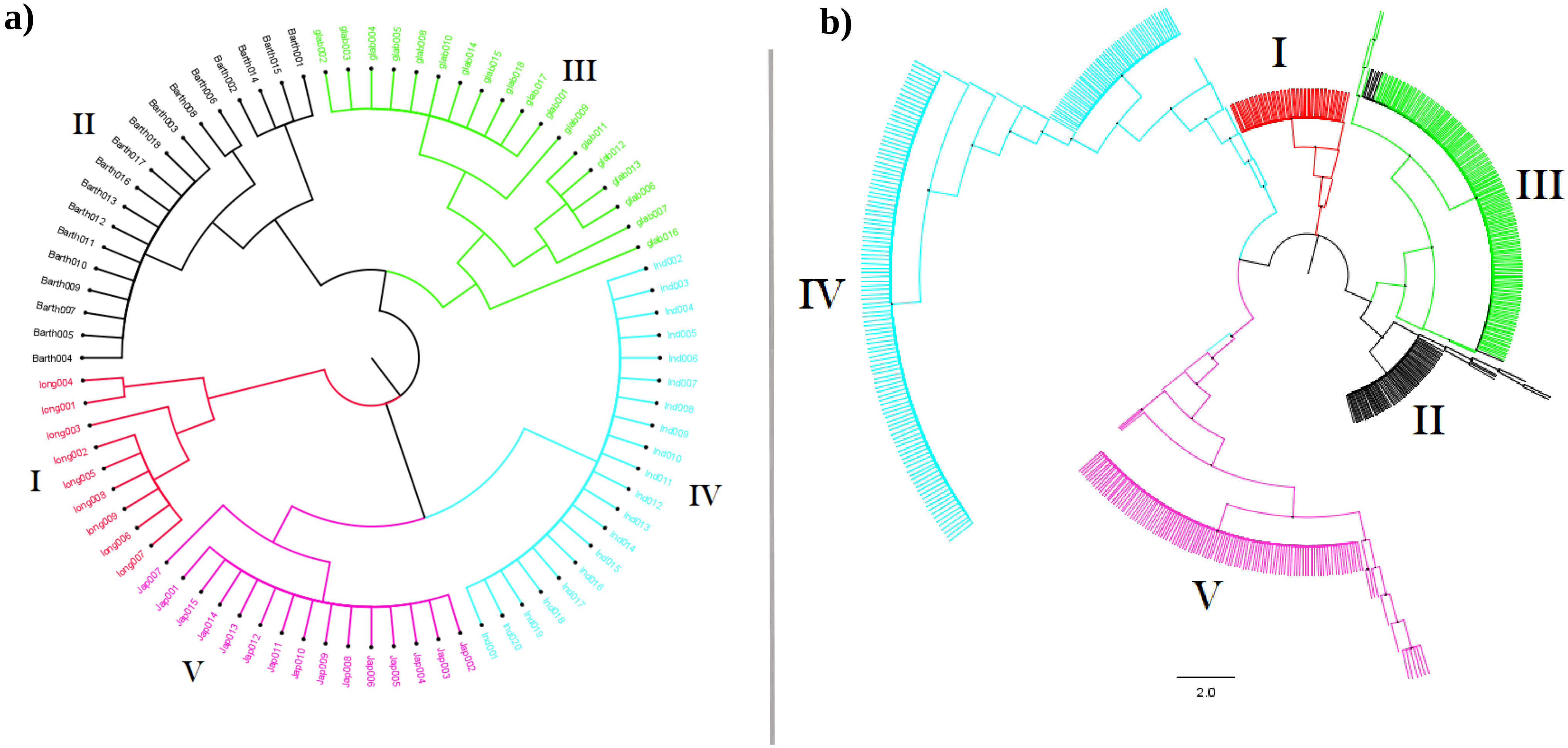

Supplementary Figure S7

Phylogenetic tree constructed using the Neighbor-Joining method. **(a)** NJ-tree based on 80 rice accessions plotted with 24 KASP SNPs diagnostic markers. **(b)** NJ-tree of 625 rice accessions using 24 KASP SNPs diagnostic markers. The colors in the tree correspond to subpopulations. Cluster **I** in red represents *O. longistaminata* accessions, cluster **II in** black represents *O. barthii* accessions, cluster **III** in green represents African rice *O. glaberrima* accessions, cluster **IV** in blue represents Asian rice *O. sativa* spp *indica* accessions, and cluster **V** in pink represents Asian rice *O. sativa* spp *japonica*
